# Correlation of the Apparent Diffusion Coefficient (ADC) with the Standardized Uptake Value (SUV) in Lymph Node Metastases of Non-Small Cell Lung Cancer (NSCLC) Patients Using Hybrid 18F-FDG PET/MRI

**DOI:** 10.1371/journal.pone.0116277

**Published:** 2015-01-09

**Authors:** Benedikt Michael Schaarschmidt, Christian Buchbender, Felix Nensa, Johannes Grueneien, Benedikt Gomez, Jens Köhler, Henning Reis, Verena Ruhlmann, Lale Umutlu, Philipp Heusch

**Affiliations:** 1 Univ Dusseldorf, Medical Faculty, Department of Diagnostic and Interventional Radiology, Dusseldorf, Germany; 2 Univ Duisburg-Essen, Medical Faculty, Department of Diagnostic and Interventional Radiology and Neuroradiology, Essen, Germany; 3 Univ Duisburg-Essen, Medical Faculty, Department of Nuclear Medicine, Essen, Germany; 4 Univ Duisburg-Essen, Medical Faculty, Department of Medical Oncology, Essen, Germany; 5 Univ Duisburg-Essen, Medical Faculty, Institute of Pathology, Essen, Germany; Uniformed Services University, UNITED STATES

## Abstract

**Objective:**

To compare the apparent diffusion coefficient (ADC) in lymph node metastases of non-small cell lung cancer (NSCLC) patients with standardized uptake values (SUV) derived from combined 18F-fluoro-deoxy-glucose-positron emission tomography/magnetic resonance imaging (18F-FDG PET/MRI).

**Material and Methods:**

38 patients with histopathologically proven NSCLC (mean age 60.1 ± 9.5y) received whole-body PET/CT (Siemens mCT™) 60min after injection of a mean dose of 280 ± 50 MBq 18F-FDG and subsequent PET/MRI (mean time after tracer injection: 139 ± 26 min, Siemens Biograph mMR). During PET acquisition, simultaneous diffusion-weighted imaging (DWI, b values: 0, 500, 1000 s/mm²) was performed. A maximum of 10 lymph nodes per patient suspicious for malignancy were analyzed. Regions of interest (ROI) were drawn covering the entire lymph node on the attenuation-corrected PET-image and the monoexponential ADC-map. According to histopathology or radiological follow-up, lymph nodes were classified as benign or malignant. Pearson’s correlation coefficients were calculated for all lymph node metastases correlating SUV_max_ and SUV_mean_ with ADC_mean_.

**Results:**

A total of 146 suspicious lymph nodes were found in 25 patients. One hundred lymph nodes were eligible for final analysis. Ninety-one lymph nodes were classified as malignant and 9 as benign according to the reference standard. In malignant lesions, mean SUV_max_ was 9.1 ± 3.8 and mean SUV_mean_ was 6.0 ± 2.5 while mean ADC_mean_ was 877.0 ± 128.6 x10^-5^ mm²/s in PET/MRI. For all malignant lymph nodes, a weak, inverse correlation between SUV_max_ and ADC_mean_ as well as SUV_mean_ and ADC_mean_ (r = -0.30, p<0.05 and r = -0.36, p<0.05) existed.

**Conclusion:**

The present data show a weak inverse correlation between increased glucose-metabolism and cellularity in lymph node metastases of NSCLC patients. 18F-FDG-PET and DWI thus may offer complementary information for the evaluation of treatment response in lymph node metastases of NSCLC.

## Introduction

Diagnostic imaging is an important pillar of clinical staging in non-small cell lung cancer (NSCLC) patients. 18F-FDG PET/CT provides sensitive detection of locoregional lymph node involvement and distant metastases [1–4] as well as high-resolution morphological CT-information, which makes PET/CT the modality of choice for TNM-Staging in potentially curable lung cancer [5–7].

However, it has been demonstrated that the combination of morphologic and functional MR-sequences (like diffusion-weighted imaging (DWI)) can be as accurate as 18F-FDG PET for whole body staging [8, 9] in NSCLC patients. In the thorax, DWI facilitates lesion detection and its information about tumor cellularity can be used to differentiate benign from malignant pulmonary masses [10–12]. For the prediction of therapy response, Ohno et al. recently showed that ADC-values derived from DWI are better suited than standardized uptake values (SUV)[13]. Although new, morphological MR-sequences still suffer from the restricted depiction of pulmonary lesions smaller than 5mm [14], morphologic MRI is advantageous in order to detect mediastinal and pleural involvement based on its high soft-tissue contrast [15].

Combining the sensitivity of PET, the accuracy of morphologic MRI and the functional information derived from DWI in a single examination could increase the diagnostic accuracy in NSCLC patients. Therefore, the recent introduction of hybrid PET/MRI-scanners could mark a turning point [16, 17]. First publications indicate that 18F-FDG PET/CT and 18F-FDG PET/MRI show a similar performance in local tumor staging as well as in the detection of nodal and distant metastases in thoracic malignancies [18–20]. As compared with morphology alone, DWI might supplement the metabolic information derived from a PET-examination with additional information regarding tumor cellularity.

Although the benefit of a simultaneous acquisition of both parameters still has to be assessed thoroughly, their combination might increase diagnostic accuracy in primary staging and could also be useful for prognostic evaluation concerning treatment response and outcome. As lymph node stage is the most important prognostic factor in NSCLC patients without distant metastases [21], improved detection of locoregional lymph node metastases and prognostic evaluation using simultaneous PET and DWI is desirable.

Therefore, the aim of this study was to correlate ADC-values and tracer uptake of thoracic lymph node metastases in order to provide a basis for the future use of PET/MRI including DWI as a tool for lymph node metastases evaluation in NSCLC.

## Material and Methods

### Patients & inclusion criteria

All patients with histologically proven NSCLC that received a whole-body 18F-FDG PET/CT and a subsequent, dedicated PET/MRI were included in this retrospective study. Patients had to be therapy naive without prior radio- or chemotherapy. The study was approved by the local ethics committee and written informed consent was obtained from all patients.

### PET/CT Imaging

All patients received a whole-body (head to upper thighs) PET/CT scan performed on a Biograph mCT (Siemens Healthcare, Erlagen, Germany) 58 ± 11 min minutes after the injection of 280 ± 50 MBq 18F-FDG. At the time of injection, blood glucose levels were below 150mg/dl. Patients were examined in low- (n = 11) and full dose (n = 27) technique: Full-dose scans were performed 70s after the injection of a contrast agent (100ml Ultravist, Bayer Schering Pharma, Berlin, Germany). Slice thickness was 5mm. For dose reduction, the manufacturer-supplied solutions CareKV and CareDose 4D were applied (presets: 120kV and 210mAs). For low-dose scans, slice thickness was 5mm and CareKV and CareDose 4D were also used (presets: 120kV and 40mAs). In general, PET-data were acquired in 7 bed positions (2min per bed position). Iterative reconstruction was performed (3 iterations, 21 subsets), a Gaussian filter of 4mm was applied. For attenuation correction, the portalvenous phase was used in full-dose scans and the low-dose CT-data in low-dose scans.

### PET/MR Imaging

A whole body PET/MRI scan was performed on a Biograph mMR (Siemens Healthcare, Erlagen, Germany). The mean waiting time after tracer injection for the PET/MRI examination was 139 ± 26min. The field of view covered an area from head to thighs. For each bed position, the following MRI-sequences were performed:

For DIXON based attenuation correction, a coronal 3D-VIBE sequence (TR 3.6ms, TE1 1.23ms, TE2 2.46ms, slice thickness 3.12mm, FOV 500×328 mm, matrix size 192×121, mm, voxel size: 4.1×2.6×3.1) was acquired. For diffusion weighted imaging, a transverse thoracic echo planar imaging (EPI) sequence (TR 17900ms, TE78ms, slice thickness 5mm, FOV 450×383mm, matrix size 160×120, mm, voxel size: 3.8×2.8×5.0, two averages) with three b-values (0, 500, 1000) in free breathing was performed. For diagnostic thoracic imaging, a coronal T2-weighted steady state free precession (TrueFISP), a transverse T1-weighted fast low angle shot sequence (FLASH) and transverse T2 half fourier acquired single shot turbo spin echo sequence (HASTE) was performed. For whole body imaging, a T1-weighted fast low angle shot sequence (FLASH) after contrast administration with fat saturation (fs) and a coronal turbo inversion recovery magnitude sequence (TIRM) was acquired (see Table 1 for further information). For all sequences, parallel imaging (GRAPPA, acceleration factor 2) was used.

**Table 1:** Sequence parameters for the diagnostic MR-sequences used in PET/MRI for NSCLC patients.

**Name**	**Region**	**Orientation**	**TR (ms)**	**TE (ms)**	**Matrix size**	**Slice thickness (mm)**	**FOV (mm)**	**Voxel size (mm)**
T2 TrueFISP	thorax	coronal	3.75	1.64	320×272	6	330×330	1.2×1.0×6.0
T2 BLADE TSE (breath hold)	thorax	transverse	4360	160	384×384	5	400×400	1.0×1.0×5.0
T1 FLASH	thorax	transverse	1510	2.15	320×256	5	400×325	1.6×1.3×5.0
T2 HASTE	thorax	coronal	649	51	320×288	6	330×330	1.1×1.0×6.0
T1 FLASH (after contrast injection, T1 = 1200)	WB	transverse	1700	3.33	256×205	7.5	450×366	2.20×1.43×7.5
TIRM (TI = 220ms)	WB	coronal	3190	55	384×288	5	450×338	1.6×1.2×5.0

In 17 patients without distant metastases in PET/CT, the protocol had to be shortened to increase patient comfort Here, additional whole-body sequences were omitted and a coronal T2-weighted BLADE TSE and a transverse T1-weighted FLASH sequence after contrast administration of the thorax was acquired instead.

PET-data were acquired in list mode (five bed positions for whole body staging: 20 min for the thorax, 4min for all other bed positions). 3D-iterative image reconstruction was performed (3 iterations, 21 subsets), a Gaussian filter of 4mm was applied. Matrix size was 344×344, voxel size was 2.01×2.01×2. During PET-acquisition, no respiratory gating was used.

Monoexponential ADC-Maps were computed by using the manufacturer-supplied software by Siemens on the corresponding PET/MRI console (Siemens Healthcare, Erlagen, Germany) directly after the examination.

### Image Analysis

**Figure 1:**
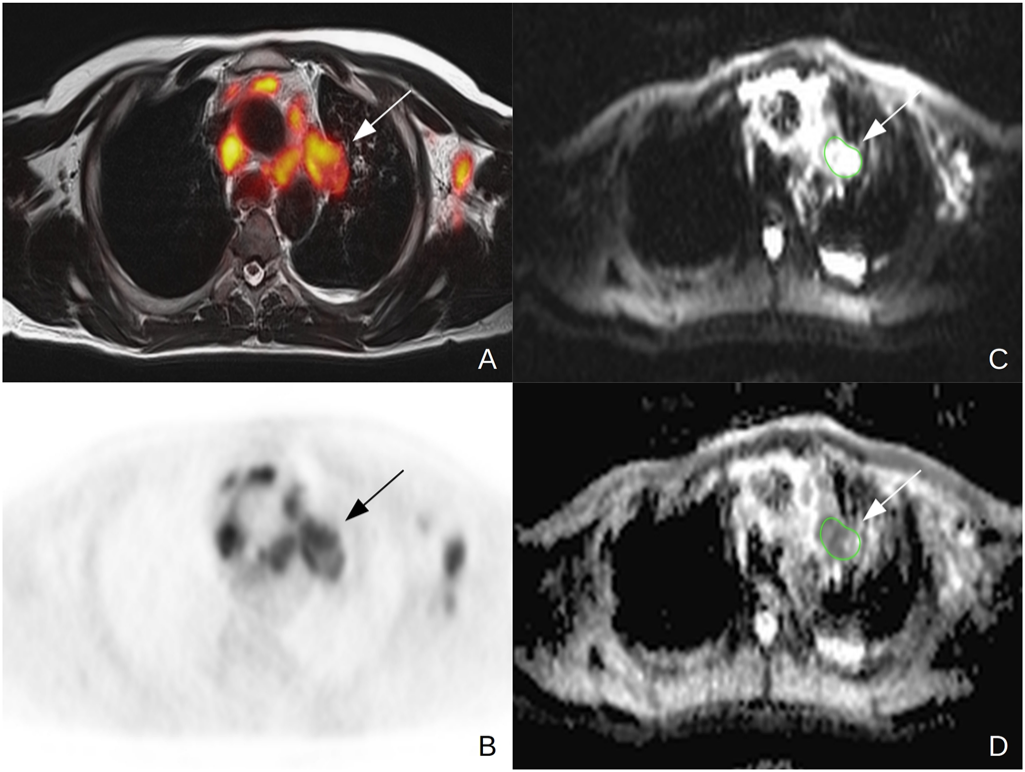
44y old male patient suffering from NSCLC (tumor stage IV, adenocarcinoma, unknown tumor grading) Note the FDG-avid lymph node in the aortopulmonary window in the fused PET/MR (A) and PET (B) images. For ADC_mean_ evaluation, a polyginal ROI was drawn around the lymph node on the b0 image in DWI and then copied to the ADCmap.

**Figure 2:**
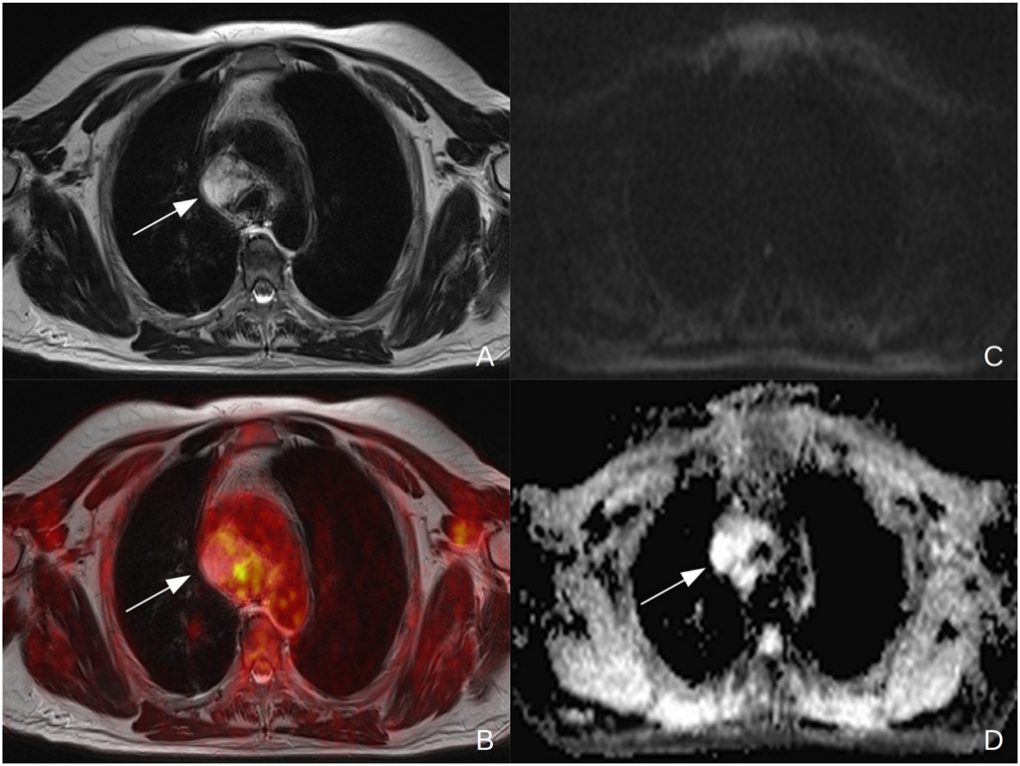
55y old male patient suffering from NSCLC (tumor stage IV, adenocarcinoma, G2) Central necrotic lymph node in the mediastinum (see arrow) with a high T2 signal in MRI (A) and a low FDG-uptake in the fused PET/MR images (B). In DWI, no diffusion restriction can be observed (C) and a T2 shine through effect can be seen on the corresponding ADC-map (D).

All images were analyzed using a dedicated OsiriX [22] Workstation (Pixmeo SARL, Bernex, Switzerland). At first, a maximum of ten malignant lymph nodes according to radiological criteria were identified for each patient, starting from the diaphragm. On CT, lymph nodes were graded as malignant or benign based on their size. Determination of lymph node size was based on measurement of the short axis diameter. A short axis diameter bigger than 10mm was considered as a sign of malignancy. Central necrosis was considered as a sign of malignant tumor spread independent of lymph node size. In MRI, malignant lymph nodes were identified according to the following criteria: increased short-axis diameter (>10mm); pathological signal intensity (heterogeneous vs. homogeneous); central necrosis; shape (smooth vs. irregular); contrast enhancement; high signal on b-1000 DWI images with low signal intensity on corresponding ADC maps. In PET, focal 18F-FDG-uptake was also considered as a sign of malignancy. PET images were assessed with and without attenuation correction of the PET data to avoid false-positive findings due to attenuation-correction artefacts.If the corresponding lymph node was visible on the MR—based attenuation corrected PET image and DWI, a freehand, polygonal region of interest (ROI) was drawn around the metastasis on MR- and CT-based attenuation corrected PET images. Then, a freehand, polygonal region of interest (ROI) was drawn around the corresponding lesion on the b0-image, which was copied to the corresponding ADC-Map and manually reshaped to avoid pixel void (Fig. 1). Osirix automatically calculated SUV_max_ and SUV_mean_ in PET and ADC_mean_ in DWI. In partly included pixels, the software used subpixel interpolation. SUV_max_ and SUV_mean_ for both modalities as well as ADC_mean_ for MR-PET were recorded for each lesion. To avoid falsifications of ADC-measurements by T2 shine through artifacts by central necrotic lymph nodes, lesions with a high signal intensity on T2-weighted imaging were excluded from further analysis (Fig. 2). After image analysis, lymph nodes were classified as benign or malignant according to the standard of reference.

### Standard of reference

Histopathological workup (e.g. after endobronchial ultrasound-guided transbronchial needle aspiration (EBUS-TBNA), biopsy or resection) was performed according to institutional standards, current diagnostic criteria of the WHO/IARC [23] were applied, and staging was performed according to the TNM Classification of Malignant Tumors (7th edition)[24]. Anatomic location for all lymph nodes was available in all histopathological reports.

In cases of absent histopathology, radiological follow-up with cross sectional imaging (CT, MRI, PET/CT and PET/MRI) of at least 90 days served as the standards of reference. Lymph nodes without reference standard were excluded from further analysis.

### Statistics

Pearson’s correlation coefficients were calculated between SUV_max_ and ADC_mean_ as well as SUV_mean_ and ADC_mean_ derived from the integrated PET/MRI examinations for all malignant lymph nodes. A subgroup analysis according to tumor histology and tumor stage was performed. Bland Altman analysis was performed and Pearson’s correlation coefficients were calculated between PET/CT and PET/MRI for SUV_max_ and SUV_mean_ in all malignant lymph nodes. P<0.05 was considered as statistically significant. IBM SPSS Statistics 22^TM^ (IBM, Armonk, NY, USA) was used for statistical analysis.

## Results

Overall, an analysis of 38 patients (14 female, 24 male, mean age 60.1 ± 9.5y) suffering from histologically proven NSCLC without prior radio- or chemotherapy was performed. All patients underwent a contrast enhanced (n = 27) or low-dose (n = 11) 18F-FDG PET/CT scan and a subsequent, dedicated PET/MRI scan.

**Figure 3:**
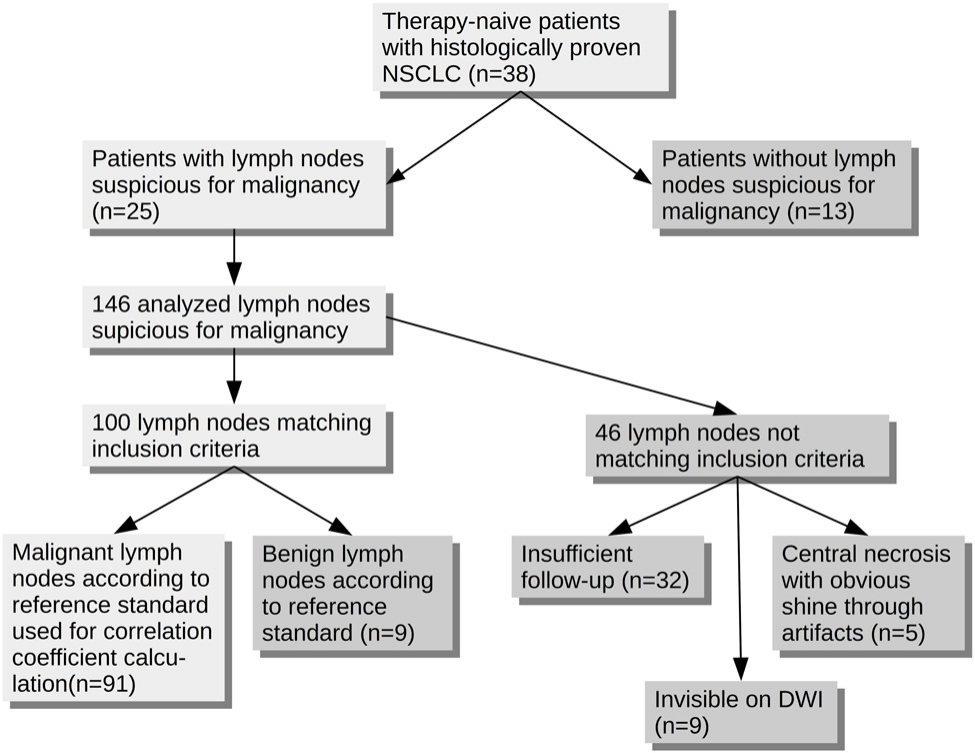
Flow-chart depicting the process of inclusion for all analyzed lymph nodes.

**Figure 4:**
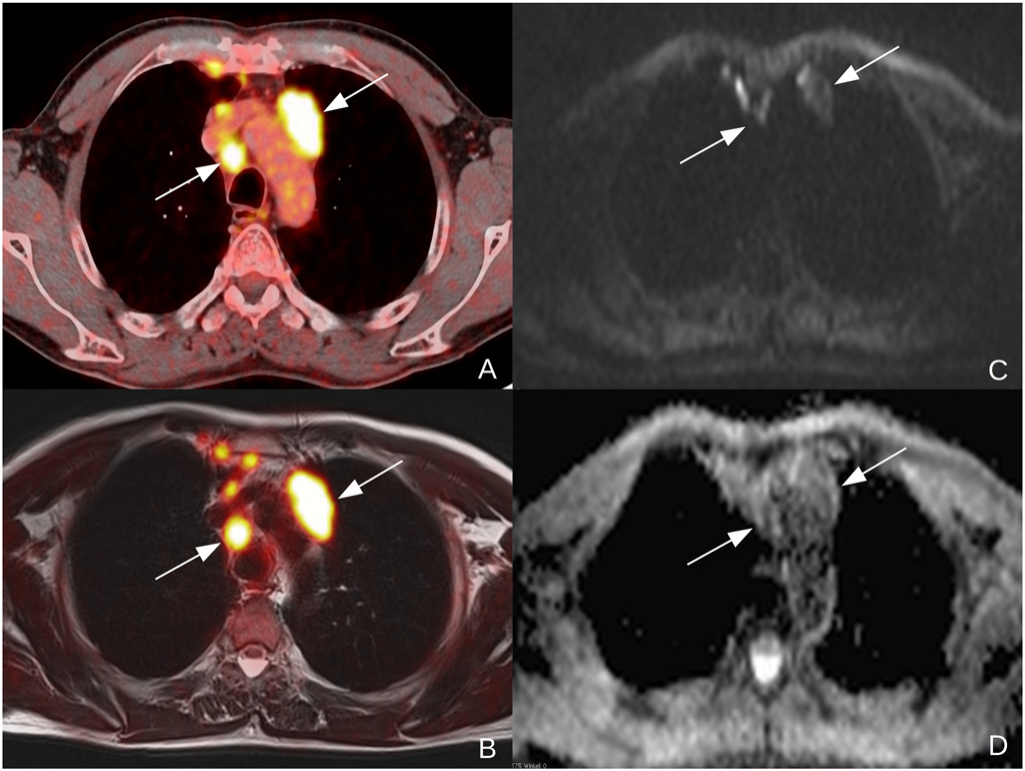
57y old male patient suffering from NSCLC (tumor stage IIIb, squamous cell carcinoma, unknown tumor grading) Clearly malignant lymph nodes to the left of the aortic arch and right to the trachea in a contrasted enhanced, fused PET/CT-image (A) and in a T2-weighted, fused PET/MR-image (B). The lymph nodes are clearly depicted in the diffusion weighted b1000-image (C) and show low ADC-values in the corresponding, monoexponential ADC-map. The additional, smaller lymph nodes in the anterior mediastinum were not visible in DWI (C) and were therefore excluded from further analysis.

A total of 146 lymph nodes suspicious for malignancy (Fig. 3) were discovered in 25 patients (adenocarcinoma: n = 16, squamous cell carcinoma: n = 6, other: n = 3, Table 2). In the 13 other patients, no suspicious lymph nodes were discovered. One patient was examined for mediastinal tumor recurrence, all other patients received PET/CT and a subsequent PET/MRI scan for initial diagnostics. One hundred lymph nodes were included in this study. Histopathological workup was available in 33 lesions, while 67 had radiological follow-up (mean time of follow-up after PET/MRI 270.5 ± 157.4 days). Forty-six lesions were excluded from analysis: In 32 lesions, no suitable standard of reference was available. Nine lymph nodes were not visible in DWI and thus could not be used for SUV-ADC correlation. Five lesions were excluded due to obvious central necrosis with shine through artifacts on the ADC-map. According to the reference standard, nine of the remaining lymph nodes were benign. Thus, FDG-PET was false-positive in these cases. Ninety-one lymph nodes were malignant (Figs. 3 & 4).

**Table 2:** Tumor characteristics in all 25 included patients with lymph nodes suspicious for malignancy in PET/CT and PET/MRI.

		**N**	**%**
Tumor stage	I	1	4
	IIA	0	0
	IIB	0	0
	IIIA	5	20
	IIIB	8	32
	IV	11	44
			
Histological subtype	Adenocarcinoma	16	64
	SCC	6	24
	other	3	12
			
Tumor grading	G1	0	0
	G2	3	12
	G3	11	44
	G4	2	8
	not available	9	36

For all 91 malignant lymph nodes, the mean SUV_max_ in PET/MRI was 9.1 ± 3.8, the mean SUV_mean_ was 6.0 ± 2.5 and the mean ADC_mean_ was 877.0 ± 128.6 x10^-5^ mm²/s while for all 9 benign lymph nodes, the mean SUV_max_ in PET/MRI was 3.3 ± 0.9, the mean SUV_mean_ was 2.6 ± 0.4 and the mean ADC_mean_ was 989.5 ± 193.3 x10^-5^ mm²/s.

**Figure 5:**
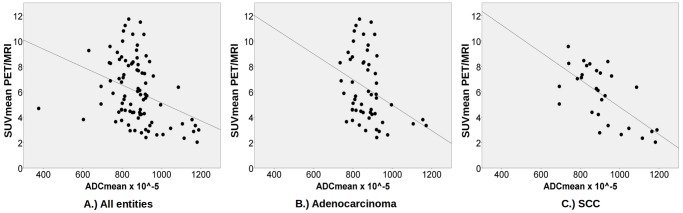
Scatter plots including the corresponding regression grade for all lymph node metastases (A, n = 91), lymph node metastases of adenocarcinoma (B, n = 54) and lymph node metastases of SCC (C, n = 29) between SUV_max_ and ADC_mean_.

For all malignant lymph nodes, the correlation between SUV_max_ and ADC_mean_ was -0.30 (p = 0.004) and between SUV_mean_ and ADC_mean_ -0.36 (p<0.001). A subgroup analysis was performed for different tumor entities and tumor stages (Table 3). Lymph node metastases in patients with squamous cell carcinoma (SCC, n = 29) showed a stronger correlation between SUV_max_ and ADC_mean_ (r = -0.55, p = 0.002) and SUV_mean_ and ADC_mean_ (r = -0.67, p<0.001) then in patients with adenocarcinoma. In this histological subtype (n = 54), r was -0.33 (p = 0.014) between SUV_max_ and ADC_mean_ and -0.34 (p = 0.012) between SUV_mean_ and ADC_mean_ (Fig. 5). In patients with stage IV disease (n = 39), no significant correlation between SUV_max_ and ADC_mean_ (r = 0.02, p>0.05) as well as SUV_mean_ and ADC_mean_ (r = -0.06, p>0.05) in metastatic lymph nodes was found. In stage IIIB disease (n = 48), r was -0.49 (p<0.001) between SUV_max_ and ADC_mean_ and -0.56 (p<0.001) between SUV_mean_ and ADC_mean_.

**Table 3:** Pearson’s correlation coefficient between SUV_max_ / ADC_mean_ and SUV_mean_ / ADC_mean_ in different subgroups of malignant lymph nodes (n = 91).

		**N**	**Correlation SUV_max_ / ADC_mean_**	**p**	**Correlation SUV_mean_ / ADC_mean_**	**p**
Tumor stage	I	0				
	II	0	*		*	
	IIIA	4	*		*	
	IIIB	48	-0.488	0.0004	-0.561	0.00003
	IV	39	0.020	0.903	-0.055	0.738
						
Histological subtype	Adenocarcinoma	54	-0.334	0.014	-0.338	0.012
	SCC	29	-0.547	0.002	-0.666	0.00008
	other	8	*		^*^	

* not performed due to small N

**Figure 6:**
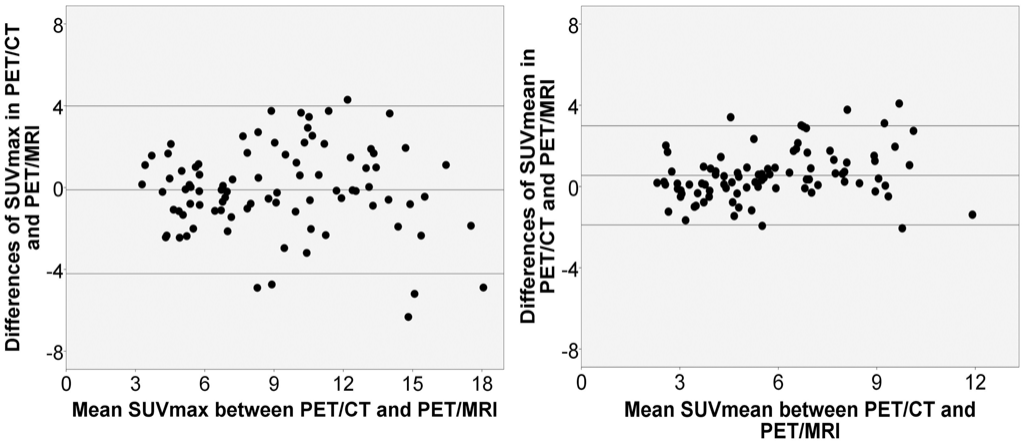
Bland-Altmann plots for SUV_max_ (Limits of agreement: 3.99 and -4.21) and SUV_mean_ (Limits of agreement: 2.99 and -1.89) between PET/CT and PET/MRI.

Bland Altmann analysis between PET/CT and PET/MRI yielded limits of agreements (LOA) of 3.99 and-4.21 for SUV_max_ and 2.99 and -1.89 for SUV_mean_ (Fig. 6). Correlation between SUV_max_ (p = 0.85, p<0.001) and SUV_mean_ (p = 0.87, p<0.001) in both modalities was strong.

## Discussion

Integrated PET/MRI is able to depict glucose metabolism and cellular density by performing simultaneous PET- and DWI-measurements. As a higher cell mass leads to an increased glucose uptake, results derived from both techniques are intertwined. Recent studies have shown a inverse correlation between ADC and SUV in primary lung cancer [25–28] as well as in lymph nodes in lymphoma patients [29]. As lymph node metastases are the most important prognostic factor in patients suffering from NSCLC, further research concerning a correlation between ADC and SUV in malignant lymph nodes was warranted. A significant inverse correlation of SUV and ADC in malignant lymph nodes in NSCLC patients is indicated by this study and, thus, supports previously published data.

Although PET and DWI are used to increase the diagnostic accuracy of morphologically unsuspicious lesions in oncological imaging, both techniques are based on different pathophysiological principles. While PET depicts glucose metabolism, DWI can be used as a marker of cellular density. As areas with an increased cellular density show an increased glucose uptake, a relation between both techniques seems plausible.

In a first evaluation performed by Regier et al. in 2011, a significant inverse correlation between SUV_max_ and ADC_min_ in primary NSCLC was found by examining sequentially acquired 18F-FDG PET/CT and MRI datasets. In simultaneous PET/MRI, these findings were corroborated by Heusch et al. for ADC_mean_ [27, 28] and Schmidt et al. for ADC_min_ [26]. For malignant lymph nodes, literature is scarce: Wu et al [29] found a weak but significant, inverse correlation between ADC and SUV in lymph node bulks of lymphoma patients. To this date, a correlation analysis between tracer uptake and ADC-values of lymph node metastases of NSCLC patients has not been performed. According to our data, a weak but significant inverse correlation in metastatic lymph nodes was demonstrated between SUV_max_ and ADC_mean_ as well as SUV_mean_ and ADC_mean_. These results are comparable to the findings of Rakheja et al.[30]. In a cohort of mixed primary malignancies, they found a weak correlation between SUV_max_ and ADC_min_, but no significant correlation for SUV_max_ and ADC_mean_.

Furthermore, no significant correlation between SUV_max_ and ADC_min_ for lymph node metastases in cervical cancer was reported in a recent study by Grueneisen et al, albeit a strong correlation between both values in primary cervical cancer was found [31]. This indicates that both values are linked due to their pathophysiological dependency, but display two different sides of tumor biology.

This correlation seems to be influenced also by the histological subtype of the tumor. Different mean ADC-values between different histological subtypes in lung cancer have already been described by Matoba et al.[32]. This seems to lead to different correlation coefficients. Regier et al. showed that especially in SCC, a strong, inverse correlation between SUV_max_ and ADC_min_ exists while it is considerably weaker in adenocarcinoma [25]. Accordingly, our data suggests a strong, significant correlation between ADC and SUV in lymph node metastases of SCC. The weaker correlation in lymph node metastases of adenocarcinoma patients (especially between SUV_mean_ and ADC_mean_) indicates that different correlations in different histological subtypes can be found also in thoracic lymph node metastases. The different correlations could be caused by histological differences between both subtypes. While small, tightly packed cells are a common histological feature in SCC, cells in adenocarcinoma are usually larger. The higher cell mass in SCCs could lead to lower ADC-values and an increased glucose uptake compared to adenocarcinomas.

Tumor stage also influences the correlation of tracer uptake and diffusion restriction: While a moderate correlation was observed in stage IIIB disease, no significant correlation was found in patients with stage IV disease. A possible explanation might be that local necrosis in higher tumor stages is more frequently present. The increased presence of free water in the tumor tissue leads to an increased mobility of water molecules and a reduced diffusion restriction on the ADC-map. As this takes place on a cellular level, a morphologically visible central necrosis cannot be detected. Still, glucose metabolism of adjacent, vital tumor cells remains high, leading to fewer changes in SUV and could therefore cause the disappearance of the correlation between ADC and SUV in higher tumor stages.

Despite the options of DWI and PET, both techniques still suffer from a lack of standardization. In DWI, first studies show a strong intrapatient as well as a moderate interscanner repeatability on scanners by different vendors and different field strengths [33–35]. In hybrid imaging however, a quantitative comparison of PET-data acquired on a PET/CT- and a PET/MRI-scanner still proves to be difficult. In both modalities, totally different techniques are used to compute an attenuation-corrected image. While the corresponding CT-scan can be used for nearly perfect attenuation correction in PET/CT, this is not possible in integrated PET/MRI. Here, the attenuation correction is based on a segment model derived from a whole-body 3D Dixon sequence under omission of skeletal attenuation [36, 37]. Therefore, discrepancies in SUV derived from PET/CT and PET/MRI found in this study are most likely attributed to technical differences in CT- and MR-based attenuation correction [38, 39]. Different time-points (approximately one and two hours after tracer injection) between the two examinations can also lead to different SUVs in lymph nodes in PET/CT and PET/MRI due to tracer elimination, different absolute distribution and decay, as recently shown by Cheng et al.[40].

This study has some limitations. Patients with a high tumor stage were overrepresented due to the focus on lymph node metastases in this study. No significant correlation was observed in patients suffering from stage IV disease, therefore, correlations might have been influenced by this patient group.

Patient numbers were not high enough for tumor grades G1 and G2. Only in patients with a G3-grade tumor, a sufficient number of examined lymph nodes was available. Hence, a comparison of correlations between SUV_max_/SUV_mean_ and ADC_mean_ according to tumor grading was not possible. The three tumor grades G1—G3 differ significantly in their cellular structure, therefore, ADC is most likely influenced by this factor as well [32]. Further studies dealing with this subject would expedite the evaluation of DWI in NSCLC.

Although DWI is a standard tool in every day clinical practice, its application in the thorax is still challenging. As image acquisition cannot be performed in a single breath hold, it has to be performed in free breathing. Motion artifacts by heartbeats, aortic pulsation and esophageal peristalsis are worsened by respiratory movement. Therefore, especially small lymph nodes cannot be depicted with DWI [41] and were excluded from this study.

The use of different PET/CT protocols (contrast-enhanced and low-dose examinations) has to be considered a further limitation. But as this analysis was mainly focused on the investigation of a possible correlation between SUV and ADC in integrated PET/MR, this does not alter the main results of this study.

In conclusion, this study shows a statistically significant inverse correlation between SUV_max_ and ADC_mean_ as well as SUV_mean_ and ADC_mean_ in lymph node metastases in NSCLC. As 18F-FDG PET and DWI depict different, but intertwined pathophysiological processes in tumor biology, both modalities could provide complementary information about lymph node metastases in NSCLC. Although a sequential acquisition is possible, the posibility to acquire both parameters simultaneously in integrated 18F-FDG PET/MRI increases patient comfort and improves clinical workflow and facilitates its introduction into clinical practice. Hence, further examinations of both functional imaging methods as tools for treatment monitoring or prognostic evaluation in lymph node metastases of NSCLC patients are warranted.

## Supporting Information

S1 File Original data of the present study. (XLS)
