# Correction: Correlation of the Apparent Diffusion Coefficient (ADC) with the Standardized Uptake Value (SUV) in Lymph Node Metastases of Non-Small Cell Lung Cancer (NSCLC) Patients Using Hybrid 18F-FDG PET/MRI

**DOI:** 10.1371/journal.pone.0120606

**Published:** 2015-03-20

**Authors:** The PLOS ONE Staff

The fourth author’s name is spelled incorrectly. The correct name is: Johannes Grueneisen.

The correct citation is: Schaarschmidt BM, Buchbender C, Nensa F, Grueneisen J, Gomez B, Köhler J, et al. (2015) Correlation of the Apparent Diffusion Coefficient (ADC) with the Standardized Uptake Value (SUV) in Lymph Node Metastases of Non-Small Cell Lung Cancer (NSCLC) Patients Using Hybrid 18F-FDG PET/MRI. PLoS ONE 10(1): e0116277. doi:10.1371/journal.pone.0116277

## References

[1] Schaarschmidt BM, Buchbender C, Nensa F, Grueneien J, Gomez B, Köhler J, et al (2015) Correlation of the Apparent Diffusion Coefficient (ADC) with the Standardized Uptake Value (SUV) in Lymph Node Metastases of Non-Small Cell Lung Cancer (NSCLC) Patients Using Hybrid 18F-FDG PET/MRI. PLoS ONE 10(1): e0116277 doi: 10.1371/journal.pone.0116277 25574968 10.1371/journal.pone.0116277 PMC4289066

